# Investigation of Knowledge, Attitude and Practice of Personal Protection Among Different Types of Workers Returning to Work Under COVID-19 Epidemic

**DOI:** 10.3389/fpubh.2021.679699

**Published:** 2021-05-17

**Authors:** Zhaoya Fan, Yuanlin Mou, Rui Cheng, Yong Zhao, Fan Zhang

**Affiliations:** School of Public Health and Management, Research Center for Medicine and Social Development, Collaborative Innovation Center of Social Risks Governance in Health, Chongqing Medical University, Chongqing, China

**Keywords:** COVID-19, knowledge, attitudes, practices, personal protection

## Abstract

**Background:** Since the outbreak of the coronavirus disease 2019 (COVID-19) world pandemic, it has had a significant negative impact on the economy and employment. The orderly resumption of work and production is an important factor in reducing the impact of the COVID-19 and an important guarantee of social and economic stability. The study aimed to investigate the knowledge, attitudes, and practice (KAP) of people returning to work about personal protection under the COVID-19 world pandemic.

**Methods:** During March 2020, based on WeChat, QQ and other internet platforms, online questionnaire survey was conducted by the convenience sampling method. SPSS version 20.0 (SPSS, Inc., Chicago, IL, USA) was used for statistics analysis. Descriptive statistics and multiple linear regression analyses were conducted to analyze the data.

**Results:** A total of 302 valid questionnaires was collected, and the valid response rate was 86.7%. About knowledge, people who return to work had the highest awareness rate of safe communication distance and the lowest awareness rate of exposure risk levels in different workplaces. The average scores of respondents in different occupations were higher than 95 in terms of personal protective attitude. In terms of practice, the average scores of respondents in different occupations were higher than 90 points. Multiple linear regression results showed that education and place of residence were the influencing factors of knowledge, while gender was the influencing factor of practice.

**Conclusion:** The awareness of prevention and control among the 302 participants was good. There were differences in personal protection knowledge among different occupational groups, but there were no differences in attitude and practice. Our findings were of great significance to improve the pertinence of COVID-19 prevention programs.

## Introduction

Since the end of December 2019, coronavirus disease (COVID-19) has spread rapidly in various regions of the world, and has become an issue of concern (1, 2). On January 30, 2020, the World Health Organization (WHO) announced that the world pandemic was listed as a public health emergency of international concern and called on all countries to fight against the epidemic (3, 4). The disease has high infectiousness, and its main clinical symptoms include fever, dry cough, fatigue, muscle pain and dyspnea (2). As of April 13, 2021, 221 countries around the world have reported 137,265,460 confirmed cases and 2,958,863 confirmed deaths from COVID-19 (5). The world pandemic has disrupted the daily work and life of the public, and the global economy has been severely damaged. It is clear that COVID-19 has become a global public health issue. In order to reduce the impact of COVID-19 outbreak, China has actively adopted medical measures and social evacuation. For example, all staff except necessary occupations should stop working before February 9, 2020 to avoid population gathering due to work (6). This measure greatly curbed the spread of the virus (7). Studies have shown that, in the environment outside the hospital, the workplace may be the best breeding ground for the virus (8). Preventive measures in the workplace can have a significant impact on the spread of a pandemic disease (9). However, studies predicted that the best time to resume work in China would be in early April. If the restrictions were lifted earlier might lead to an earlier and higher second peak (10). Therefore, in the face of the severity of the world pandemic, it is important to take relevant protective measures for those who return to work in February and March. On March 5, news.cn/worldpro/ issued a series of guidelines related to returning to work: indoor ventilation and disinfection should be done well in the workplace; employers should establish health records of employees and do not discriminate against employees confirmed or suspected of being infected with COVID-19; employees should insist on wearing masks and keeping a safe distance from colleagues, etc. (11). Specifically, farmers can carry out agricultural activities on a staggered peak and time sharing to avoid unnecessary gathering and contact. For the general staff, try not to take public transport when going out, consciously accept temperature monitoring when entering the unit, and keep the office clean and hygienic. If conditions permit, the company can allow employees to telecommute from home to avoid the risks brought by commuting, and at the same time pay close attention to their daily physical conditions. It is more important for high-risk medical workers to do a good job of self-protection. At ordinary times, it is necessary to strengthen the disinfection of the environment, prevent nosocomial infection, establish a strict ward management system, control the entry and exit of irrelevant personnel, reduce the frequency and time of family visits, wear protective clothing, goggles and gloves correctly during work (12, 13). In addition, special attention should be paid to the adjustment of negative emotions. Studies have shown that medical workers are more likely to have psychological problems (14, 15).

So far, the global epidemic prevention and control is still in a critical period, and some areas have been in a state of resumption of work. It is worth noting that during the period of resuming work and increasing production, the effect of prevention and control of pneumonia is greatly affected by the knowledge, attitudes and practices (KAP) of the people returning to work. The level of knowledge of a disease can affect people's attitudes and practices, on the other hand, negative attitudes and practices can increase the risk of death from disease (16, 17). Therefore, the purpose of this study is to assess the KAP status quo and explore the possible influencing factors of different occupational groups, so as to provide valuable information for countries and regions that have returned to work or are ready to return to work.

## Materials and Methods

### Study Design and Sampling

This cross-sectional study was conducted on a professional questionnaire survey platform (https://www.wenjuan.com) from March 23 to April 8, 2020. Convenience sampling method was used to select participants. According to the Kendall sample estimation method for multivariate analysis, the sample size was required to be 5–10 times the number of variables (18). In our survey, a total of 6 basic information items and 41 questionnaire dimensions were covered, therefore the minimum sample size of this survey was 235–470. Those returning to work who can understand the contents of the questionnaire and can access to the internet were eligible to participate. Finally, 348 people completed the online questionnaire, 302 of which were valid. We also performed *post hoc* power analysis using G-power 3.1.9.7 (Kiel University, Kiel, Germany) software to test the power of our study. The power (1-β) was determined to be 0.996, based on an error probability (significance level) of 0.05, and total sample size of 302.

### Measures and Analysis

#### Instrument

The questionnaire was designed on the basis of guidelines for COVID-19 prevention and control issued by the WHO (19), the National Health Commission of the People's Republic China (20), the Chinese Center for Disease Control and Prevention (21). The relevant experts were invited to discuss the questionnaire to make the questions and options as accurate and comprehensive as possible. Cronbach's alpha for the questionnaire total was 0.951 which indicated that the internal consistency of the questionnaire was good. The questionnaire was a voluntary and anonymous survey. No private information and sensitive language. The questionnaire consisted of four sections: demographic variables, knowledge, attitudes and practices with respect to COVID-19.

Demographic variables (six items): age, gender, education, occupation, etc. For occupations, it involved more than a dozen industries. In order to avoid too few people in each category, we divided the occupations into four groups: (1) white-collar workers (including officials, public servants, managers, office clerks, etc.); (2) professionals (including doctors, nurses, teachers, reporters, lawyers, etc.); (3) blue-collar workers (including mill workers, waiters, salespeople, etc.); (4) self-employments (including contractors, freelancers, farmers, herdsmen, fishermen, etc.). As for the self-filled occupation questionnaires, we classified them into the above four categories according to the Code of Occupational Classification of the People's Republic of China (2015 Edition).

Knowledge of personal protection (10 items): infectious disease classification, measures to inactivate the virus, the source of infection, the main route of transmission, susceptible population, daily protection knowledge, safe communication distance, risk of infection in different workplaces, mask selection, anti-virus measures after going home. There were three options for each item, one point was given for a correct answer. The higher the score, the better the knowledge.

Attitude of personal protection (seven items): actively guard against virus, attitude toward confirmed cases around them, cooperate with the protective measures of the work unit, etc. Each item used the Likert scale to assess the degree of agreement with the statement, with options ranging from 1 (strongly disagree) to 5 (strongly agree). The total score was converted into a percentage system, the higher the score, the more positive attitude.

Practice of personal protection (24 items): pay attention to world pandemic information, wear masks, avoid crowds gathering, keep the indoor ventilation, etc. It involved the protective practice in four aspects: at home, commuting, working and dining behavior. Each item used the Likert scale to assess the degree of agreement with the statement, with options ranging from 1 (strongly disagree) to 5 (strongly agree). The total score was converted into a percentage system, with higher scores indicating better practice.

#### Data Analysis

We described the characteristics of participants based on four groups according to their occupations, the results were presented as frequencies and percentages. The KAP scores of different occupational groups were presented as means and interquartile rage. Mann-Whitney U test and Kruskal–Wallis *H* test were used to analyze the differences of KAP among different occupational groups. A multiple linear regression model was used to identify factors associated with KAP. *P* < 0.05 (two-tailed) was considered statistically significant. All statistics analyses were performed with SPSS version 20.0 (SPSS, Inc., Chicago, IL, USA).

## Results

### Basic Characteristics of the Respondents

Table 1 presented the basic characteristics in the whole sample. A total of 348 questionnaires was collected in this survey. After excluding duplicate and invalid questionnaires, 302 were enrolled, and the effective response rate was 86.7%. Most of the respondents were female (56.1%), urban residents (97.6%), with an educational level of undergraduate or junior college (74.4%). Among those who returned to work in different occupations, white-collar workers, professionals, blue-collar workers and self-employments were 82 (27.2%), 113 (37.4%), 46 (15.2%), and 61 (20.2%), respectively.

**Table 1:** Demographic characteristics of respondents.

	**White-collar workers (%)**	**Professionals (%)**	**Blue-collar workers (%)**	**Self-employments (%)**	**Total (%)**
**Gender**							
Male	36 (43.9)	28 (24.8)	26 (56.5)	27 (44.3)	117 (38.7)
Female	46 (56.1)	85 (75.2)	20 (43.5)	34 (55.7)	185 (61.3)
**Age (years)**							
15-30	25 (30.5)	41 (36.3)	17 (37)	42 (68.9)	125 (41.4)
31-45	29 (35.4)	43 (38.1)	14 (30.4)	10 (16.4)	96 (31.8)
46-60	28 (34.1)	29 (25.7)	15 (32.6)	9 (14.8)	81 (26.8)
**Education**							
Senior high school and below	12 (14.6)	7 (6.2)	34 (73.9)	18 (29.5)	71 (23.5)
Undergraduate and junior college	61 (74.4)	88 (77.9)	12 (26.1)	42 (68.9)	203 (67.2)
Master degree or above	9 (11)	18 (15.9)	0 (0)	1 (1.6)	28 (9.3)
**Place of residence**							
Urban	80 (97.6)	103 (91.2)	29 (63)	37 (60.7)	249 (82.5)
Rural	2 (2.4)	10 (8.8)	17 (37)	24 (39.3)	53 (17.5)
Total	82 (27.2)	113 (37.4)	46 (15.2)	61 (20.2)	302 (100.0)

### COVID-19 Knowledge

Table 2 showed the options and responses for each question, of the 302 respondents, measures to inactivate the virus (96.4%), susceptible population (90.1%), and anti-virus measures after going home (94.7%) had a higher scoring rate. However, the scoring rates of infectious disease classification (45.0%), route of transmission (56.3%), and mask selection (47.0%) were lower. The highest (safe communication distance) and lowest (risk of infection in different workplaces) item scoring rates were 99.0 and 10.9%, respectively. Among those who answered correctly, the chi-square test was used to compare what aspects of knowledge were different among people returning to work in different occupations, and the results showed that there were statistical differences in infectious disease classification (χ^2^ = 53.658, *P* < 0.001), the source of infection (χ^2^ = 23.883, *P* < 0.001), the route of transmission (χ^2^ = 14.282, *P* = 0.003), the risk level of the workplace (χ^2^ = 8.986, *P* = 0.029) and the choice of masks (χ^2^ = 12.405, *P* = 0.006). More details were shown in Table 3.

**Table 2:** Responses to knowledge of COVID-19 among people returning to work in different occupations.

**Item**	**Response**, ***n* (%)**
**K1: What is the classification of infectious diseases in COVID-19?**	
A. Class A infectious disease	163 (54.0)
B. Class B infectious disease	136 (45.0)
C. Class C infectious disease	3 (1.0)
**K2: Which of the following measures can effectively inactivate novel coronavirus?**	
A. Chlorine disinfectants, peracetic acid, etc.	291 (96.4)
B. Smoked vinegar	4 (1.3)
C. Gargle with salt water	7 (2.3)
**K3: Which of the following is wrong about the possible source of infection of novel coronavirus?**	
A. Asymptomatic infected	43 (14.2)
B. COVID-19 patients	16 (5.3)
C. Recovered from COVID-19	243 (80.5)
**K4: Which of the following is not the main route of transmission of the novel coronavirus?**	
A. Droplet and contact transmission	35 (11.6)
B. High concentration aerosol transmission	97 (32.1)
C. Fecal-oral transmission	170 (56.3)
**K5: Which of the following is true about the susceptibility of the novel coronavirus?**	
A. People are generally susceptible	272 (90.1)
B. Old people and children are susceptible	15 (5.0)
C. People with underlying diseases are susceptible	15 (5.0)
**K6: Which of the following can enhance the protection against novel coronavirus?**	
A. Wear a multi-layer mask	59 (19.5)
B. Drink liquor	4 (1.3)
C. Wash hands and disinfect in time	239 (79.1)
**K7: Which of the following is true to contact with others in public during an outbreak?**	
A. Normal communication	2 (0.7)
B. Wear a mask and keep a distance of more than one meter	299 (99.0)
C. Wear a mask	1 (0.3)
**K8: What is the risk level of infection for staff in crowded place during the epidemic?**	
A. High risk exposure	266 (88.1)
B. Medium risk exposure	33 (10.9)
C. Low risk exposure	3 (1.0)
**K9: What kind of mask should be chosen when there is only particulate matter (such as dust, smoke) in the workplace during the epidemic?**	
A. Disposable mask	67 (22.2)
B. Surgical mask	93 (30.8)
C. Anti-particulate mask	142 (47.0)
**K10: What are the correct anti-virus measures should be taken when going home during the epidemic?**	
A. Replace clothes, wash hands and disinfect in time	286 (94.7)
B. Place mask and clothes at will	10 (3.3)
C. Clean the mask and use it again	6 (2.0)

**Table 3:** Comparison of knowledge level of people returning to work in different occupations regarding COVID-19.

	**White-collar workers (%)**	**Professionals (%)**	**Blue-collar workers (%)**	**Self-employments(%)**	**Total (%)**	**χ^2^**	***P***
K1: What is the classification of infectious diseases in COVID-19?	27 (32.9)	80 (70.8)	7 (15.2)	22 (36.1)	136 (45.0)	53.658	<0.001^***^
K2: Which of the following measures can effectively inactivate novel coronavirus?	79 (96.3)	110 (97.3)	43 (93.5)	59 (96.7)	291 (96.4)	1.639	0.700
K3: Which of the following is wrong about the possible source of infection of novel coronavirus?	67 (81.7)	102 (90.3)	26 (56.5)	48 (78.7)	243 (80.5)	23.883	<0.001^***^
K4: Which of the following is not the main route of transmission of the novel coronavirus?	51 (62.2)	74 (65.5)	21 (45.7)	24 (39.3)	170 (56.3)	14.282	0.003^**^
K5: Which of the following is true about the susceptibility of the novel coronavirus?	78 (95.1)	101 (89.4)	38 (82.6)	55 (90.2)	272 (90.1)	5.248	0.144
K6: Which of the following can enhance the protection against novel coronavirus?	61 (74.4)	95 (84.1)	32 (69.6)	51 (83.6)	239 (79.1)	6.076	0.108
K7: Which of the following is true to contact with others in public during an outbreak?	80 (97.6)	113 (100)	45 (97.8)	61 (100.0)	299 (99.0)	3.774	0.174
K8: What is the risk level of infection for staff in crowded place during the epidemic?	7 (8.5)	20 (17.7)	3 (6.5)	3 (4.9)	33 (10.9)	8.986	0.029^*^
K9: What kind of mask should be chosen when there is only particulate matter (such as dust, smoke) in the workplace during the epidemic?	36 (43.9)	66 (58.4)	21 (45.7)	19 (31.1)	142 (47.0)	12.405	0.006^**^
K10: What are the correct anti-virus measures should be taken when going home during the epidemic?	77 (93.9)	109 (96.5)	44 (95.7)	56 (91.8)	286 (94.7)	2.000	0.583

* *P < 0.05*,

** *P < 0.01*,

*** *P < 0.001*.

### COVID-19 Attitude and Practice

The attitude scores of different occupational groups were all higher than 95, and practice scores were all higher than 90. For people of different occupations, the influencing factors of KAP were different. Education level was the influencing factor of white-collar workers practice, residence was the influencing factor of blue-collar attitude, and gender was the influencing factor of professional attitude and practice. The results of Kruskal–Wallis *H* test showed that there were differences in knowledge among people of different occupations, but not in attitudes and practices. More details were shown in Table 4.

**Table 4:** Univariate analysis of factors associated with KAP.

		**White-collar workers**	**Statistic**	**Professionals**	**Statistic**	**Blue-collar workers**	**Statistic**	**Self-employments**	**Statistic**
Knowledge	Gender		743.50^a^		1024.00^a^		222.50^a^		389.00^a^
	Male	7.00 (6.00, 8.00)		8.00 (7.00, 8.00)		6.00 (4.75, 7.00)		7.00 (6.00, 8.00)	
	Female	7.00 (6.00, 8.00)		8.00 (7.00, 9.00)		6.00 (6.00, 7.00)		6.00 (6.00, 7.00)	
	Age (years)		1.69^b^		1.54^b^		5.55^b^		2.82^b^
	15–30	7.00 (5.00, 8.00)		8.00 (7.00, 8.50)		5.00 (4.50, 6.50)		6.00 (6.00, 7.00)	
	31–45	7.00 (6.50, 8.00)		8.00 (7.00, 9.00)		7.00 (6.00, 8.00)		7.00 (6.75, 8.00)	
	46–60	7.00 (6.00, 7.75)		8.00 (7.00, 9.00)		6.00 (5.00, 7.00)		6.00 (6.00, 7.50)	
	Education		6.50^b^^*^		5.33^b^		147.50^a^		0.74^b^
	Senior high school and below	6.00 (4.00, 7.00)		7.00 (3.00, 7.00)		6.00 (5.00, 7.00)		6.50 (5.75, 7.00)	
	Undergraduate and junior college	7.00 (6.00, 8.00)		8.00 (7.00, 9.00)		5.50 (4.25, 6.75)		7.00 (6.00, 7.25)	
	Master degree or above	7.00 (7.00, 8.50)		8.00 (7.00, 9.00)		NA		6.00 (6.00, 6.00)	
	Place of residence		38.50^a^		370.50^a^		195.00^a^		363.50^a^
	Urban	7.00 (6.00, 8.00)		8.00 (7.00, 9.00)		6.00 (5.00, 7.50)		7.00 (6.00, 7.00)	
	Rural	5.00 (3.00, NA)		7.50 (6.25, 8.00)		6.00 (4.50, 7.00)		6.00 (5.00, 7.00)	
	Total	7.00 (6.00, 8.00)		8.00 (7.00, 9.00)		6.00 (5.00, 7.00)		7.00 (6.00, 7.00)	56.73^b^^***^
Attitude	Gender		822.50^a^		944.50^a^^*^		259.50^a^		441.00^a^
	Male	100.00(97.14, 100.00)		100.00 (89.29, 100.00)		100.00 (99.29, 100.00)		100.00 (94.29, 100.00)	
	Female	100.00 (96.43, 100.00)		100.00 (100.00, 100.00)		100.00 (97.86, 100.00)		100.00 (93.58, 100.00)	
	Age (years)		1.10^b^		4.36^b^		1.37^b^		1.40^b^
	15–30	100.00 (97.14, 100.00)		100.00 (97.14, 100.00)		100.00 (100.00, 100.00)		100.00 (88.57, 100.00)	
	31–45	100.00 (98.57, 100.00)		100.00 (100.00, 100.00)		100.00 (98.57, 100.00)		100.00 (99.29, 100.00)	
	46–60	100.00 (95.00, 100.00)		100.00 (100.00, 100.00)		100.00 (97.14, 100.00)		100.00 (95.72, 100.00)	
	Education		0.29^b^		3.56^b^		203.50^a^		0.77^b^
	Senior high school and below	100.00 (85.72, 100.00)		100.00 (91.43, 100.00)		100.00 (99.29, 100.00)		100.00 (94.29, 100.00)	
	Undergraduate and junior college	100.00 (97.14, 100.00)		100.00 (100.00, 100.00)		100.00 (97.86, 100.00)		100.00 (90.72, 100.00)	
	Master degree or above	100.00 (97.14, 100.00)		100.00 (98.57, 100.00)		NA		100.00 (100.00, 100.00)	
	Place of residence		54.00^a^		451.00^a^		172.50^a^^*^		437.50^a^
	Urban	100.00 (97.14, 100.00)		100.00 (100.00, 100.00)		100.00 (95.72, 100.00)		100.00 (94.29, 100.00)	
	Rural	100.00 (100.00, 100.00)		100.00 (93.58, 100.00)		100.00 (100.00, 100.00)		100.00 (90.00, 100.00)	
	Total	100.00 (97.14, 100.00)		100.00 (100.00, 100.00)		100.00 (98.29, 100.00)		100.00 (94.29, 100.00)	5.84^b^
Practice	Gender		805.50^a^		762.50^a^^**^		219.00^a^		403.50^a^
	Male	99.59(89.17, 100.00)		98.33 (84.37, 100.00)		99.59 (94.17, 100.00)		100.00 (85.83, 100.00)	
	Female	99.17(87.92, 100.00)		100.00 (98.33, 100.00)		97.09 (89.17, 100.00)		100.00 (96.46, 100.00)	
	Age (years)		0.14^b^		0.35^b^		1.04^b^		5.05^b^
	15–30	100.00 (86.25, 100.00)		100.00 (97.50, 100.00)		99.17 (95.84, 100.00)		99.17 (88.12, 100.00)	
	31–45	99.17 (90.00, 100.00)		100.00 (98.33, 100.00)		96.67 (89.58, 100.00)		100.00 (99.79, 100.00)	
	46–60	96.67 (91.67, 100.00)		100.00 (96.25, 100.00)		100.00 (86.67, 100.00)		100.00 (84.17, 100.00)	
	Education		7.75^b^^*^		2.76^b^		191.50^a^		0.80^b^
	Senior high school and below	96.25 (82.92, 99.79)		100.00 (97.50, 100.00)		99.17 (89.79, 100.00)		100.00 (85.83, 100.00)	
	Undergraduate and junior college	100.00 (92.92, 100.00)		100.00 (98.33, 100.00)		99.17 (96.04, 100.00)		99.59 (92.50, 100.00)	
	Master degree or above	91.67 (84.17, 95.00)		98.75 (91.87, 100.00)		NA		100.00 (100.00, 100.00)	
	Place of residence		36.00^a^		465.50^a^		208.50^a^		413.50^a^
	Urban	99.17 (88.33, 100.00)		100.00 (97.50, 100.00)		99.17 (89.59, 100.00)		100.00 (89.17, 100.00)	
	Rural	100.00 (100.00, 100.00)		100.00 (98.12, 100.00)		100.00 (93.75, 100.00)		100.00 (93.54, 100.00)	
	Total	99.17 (88.33, 100.00)		100.00 (97.50, 100.00)		99.17 (91.67, 100.00)		100.00 (91.67, 100.00)	7.10^b^

a *outcomes of Mann-Whitney U test*,

b *outcomes of Kruskal-Wallis H test*.

* *P < 0.05*,

** *P < 0.01*,

*** *P < 0.001*.

*Bold indicates that the results are statistically significant*.

### Multiple Linear Regression Analysis of the Factors Influencing KAP

Knowledge score was taken as the dependent variable, gender (male = 1, female = 2), age (15–30 years old = 1, 31–45 years old = 2, 46–60 years old = 3), education (senior high school and below = 1, undergraduate and junior college = 2, master degree or above = 3), place of residence (urban = 1, rural = 2), occupation (white-collar workers = 1, professionals = 2, blue-collar workers = 3, self-employments = 4), were the five independent variables. Multiple linear regression was conducted by the forced entry method to quantify the independent contributions of the above variables to knowledge. The same two models were built using the scores of attitude and practice as dependent variables. After adjusting for demographic variables, we found that the factors influencing knowledge were the level of education and place of residence, specifically reflected in living in the urban, the higher the level of education, the higher the level of knowledge. Gender was a predictor for practice, and females might have a better personal protective practice than males. More details were shown in Table 5.

**Table 5:** Multiple linear regression analysis of the factors influencing KAP.

	**Unstandardized Coefficient**		**Standardized Coefficient**	**t**	**95% CI**	**P**
	**B**	**Standard Error**	**β**			
**Knowledge**							
Gender	0.337	0.176	0.104	1.918	(−0.009, 0.683)	0.056
Age	0.182	0.112	0.094	1.625	(−0.038, 0.403)	0.105
Education	0.745	0.166	0.261	4.479	(0.418, 1.072)	<0.001^***^
Place of residence	−0.739	0.251	−0.177	−2.946	(−1.232, −0.245)	0.003^**^
Occupation	0.002	0.090	0.002	0.025	(−0.176, 0.18)	0.980
**Attitude**							
Gender	1.381	1.236	0.065	1.117	(−1.052, 3.814)	0.265
Age	0.475	0.788	0.037	0.602	(−1.076, 2.025)	0.547
Education	1.405	1.170	0.075	1.201	(−0.897, 3.707)	0.231
Place of residence	1.547	1.763	0.057	0.877	(−1.923, 5.016)	0.381
Occupation	0.500	0.636	0.052	0.786	(−0.752, 1.752)	0.433
**Practice**							
Gender	2.603	1.299	0.116	2.005	(0.047, 5.159)	0.046^*^
Age	0.951	0.828	0.071	1.148	(−0.678, 2.58)	0.252
Education	0.603	1.229	0.031	0.491	(−1.815, 3.021)	0.624
Place of residence	1.520	1.852	0.053	0.821	(−2.125, 5.165)	0.412
Occupation	0.267	0.668	0.026	0.400	(−1.048, 1.583)	0.689

* *P < 0.05*,

** *P < 0.01*,

*** *P < 0.001*.

## Discussion

Since the discovery of novel coronavirus, COVID-19 has had a destructive impact on the world. Health departments must formulate effective strategies to educate and manage the public. It is particularly important to timely grasp the current status and influencing factors of workers' KAP on epidemic prevention and control in the process of resuming work and production. Therefore, the purpose of this study was to compare the KAP regarding to COVID-19 among people returning to work in different occupations in China, which provides a theoretical reference for strengthening society-wide efforts to prevent and control the world pandemic. In this study, there were great differences in knowledge scoring rates in different aspects. Some professional issues such as risk of infection in different workplaces (K8:10.9%), infectious disease classification (K1: 45%) and mask selection in different places (K9: 47%) had low scores, suggesting these are the weak areas to be strengthened. This was different from the results of Zhong et al. (the overall correct rate of the knowledge questionnaire was 90%) (22). The reason might be that Zhong's survey area was mainly Wuhan city (where the first confirmed case was found in China), and most of the people surveyed were well-educated people (82.4% held an associate's degree or higher, and 56.2% engaged in mental labor). For the attitudes and practices about prevention and control of COVID-19 of the returning workers, with COVID-19 spread rapidly from a single city to the whole country, the vast of majority of respondents showed a high degree of compliance. The scores of attitude and practice were all higher than 95 and 90 respectively. According to the WHO, social distancing/self-isolation and lockdown were two crucial nationwide social measures during a public health crisis (23). After COVID-19 broke out across the country, the Chinese government took unprecedented measures to control the world pandemic, including quarantine and isolation, strict management of working and living spaces and the Examine and Approve Policy on the resumption of work. All occupations expressed high support for the measures taken by the government, health institutions and communities prevent and control the world pandemic.

Our study found that individual demographic characteristics had an impact on their KAP, which is consistent with previous studies (24–26). Educational level and place of residence were the influencing factors of respondents' knowledge, which was also in line with the law of low educational level in rural areas. The results suggested that people living in rural areas with a low level of education also had a lower level of knowledge. This may be due to residents in rural areas have limited availability to the internet and online health information resources and they are more likely to have poor knowledge about COVID-19. Less educated residents may be more likely to receive visual media rather than textual official documents. We suggest that more targeted health education should be carried out. For villages and towns to increase broadcasting and other more acceptable publicity methods, for people with low educational level to use more easy-to-understand popular science means such as simple cartoons. For people of different occupations, the knowledge score of professional personnel was generally higher than that of other types of workers, probably because they have received more training, have stronger learning ability, and easier to master COVID-19 's prevention and control knowledge. We also found that gender was a factor affecting practice. Females showed better protective practice, consistent with the results of a study in Vietnam (27), which may be related to the fact that males were more likely to engage in risk-taking behavior (28). Knowledge is the basis for establishing correct attitudes to change practice, while the attitude is the driving force of practice change. People's practice is influenced by their own knowledge and attitude (29). The KAP theoretical model is helpful for public health policy makers and health workers to identify the target population of COVID-19's prevention and health education.

During the outbreak of infectious diseases, timely understand the public's KAP of the epidemic and the needs of health education, and carry out effective risk communication with the public, so as to take health education measures and strategies for different groups of people. So as to control the spread of virus in the population in a timely manner. This can effectively reduce the negative psychological reaction caused by the outbreak of the world pandemic, eliminate public panic, prevent the spread of virus, which is conducive to the prevention and control of the epidemic (30). This study conducted a rapid evaluation of public KAP in the rising phase of COVID-19, which can provide a basis for the government to formulate targeted health education and behavior intervention strategies.

This study also has some limitations. Although we have designed the questionnaire according to the official guidance manual and the latest literature, the depth of the survey may be limited. Second, this study is a web-based survey, which will result in the loss of some respondents (those who do not know how to use the internet or cannot use the internet or smart phones due to restrictions), and overestimate the KAP level of those who return to work to a certain extent. Third, there may be a recall bias because the data are collected through participants' self-reports.

## Conclusion

This study preliminarily explored the KAP and influencing factors of people returning to work in different occupations on the prevention and control of COVID-19. Overall, these workers in our survey showed positive attitude and appropriate practice toward COVID-19 during the world pandemic, which are significant factors to limit the spread of the virus. The results of this survey can provide a reference for the subsequent improvement of COVID-19's prevention publicity and health education. Due to the sample limitation, more extensive studies are needed in the future to support our conclusions.

## Data Availability Statement

The raw data supporting the conclusions of this article will be made available by the authors, without undue reservation.

## Author Contributions

The manuscript writing for the original draft and data analysis were completed by ZYF. YLM collaborated in data extraction and gave the paper revision and format adjustment work. FZ and YZ gave the entire process technical and paper writing guidance support. All authors read and approved the final manuscript.

## Conflict of Interest

The authors declare that the research was conducted in the absence of any commercial or financial relationships that could be construed as a potential conflict of interest.
